# Targeted next-generation sequencing-based molecular diagnosis of congenital hand malformations in Chinese population

**DOI:** 10.1038/s41598-018-30940-6

**Published:** 2018-08-24

**Authors:** Litao Qin, Guiyu Lou, Liangjie Guo, Yuwei Zhang, Hongdan Wang, Li Wang, Qiaofang Hou, Hongyan Liu, Xichuan Li, Shixiu Liao

**Affiliations:** 1 Medical Genetic Institute of Henan Province, Henan Provincial Key Laboratory of Genetic Diseases and Functional Genomics, Henan Provincial People’s Hospital, People’s Hospital of Zhengzhou University, Zhengzhou, Henan China; 2 Department of Immunology, Tianjin Medical University, Tianjin, China

## Abstract

Congenital hand malformations is rare and characterized by hand deformities. It is highly heterogeneous, both clinically and genetically, which complicates the identification of causative genes and mutations. Recently, targeted next-generation (NGS) sequencing has been successfully used for the detection of heterogeneous diseases, and the use of NGS also has contributed significantly in evaluating the etiology of heterogeneous disease. Here, we employed targeted NGS to screen 248 genes involved in genetic skeletal disorders, including congenital hand malformations. Three pathogenic mutations located in the *GJA1*, *ROR2* and *TBX5* genes were detected in three large Chinese families with congenital hand malformations. Two novel mutations were reported, and a known causative mutation was verified in this Chinese population. This is also the first report that the same panel of targeted NGS was employed to perform molecular diagnosis of different subtypes of congenital hand malformations. Our study supported the application of a targeted NGS panel as an effective tool to detect the genetic cause for heterogeneous diseases in clinical diagnosis.

## Introduction

Congenital hand malformations characterized by hand deformities is rare and occurs in approximately 23 of 10,000 total births^1,2^. It is also a highly clinically heterogeneous disease with different phenotypes, including syndactyly (SD), brachydactyly (BD), and ectrodactyly. Moreover, a particular phenotype also contains several subtypes, such as brachydactyly (BD), a common subtype of congenital hand deformity and characterized by short fingers and toes^3^. BD contains five subtypes (Type A-E), and Type A can further be classified as five subtypes (Type A1, A2, A3, A4, and A5)^4,5^. Because of the complexity of phenotypes congenital hand malformations, an accurate diagnosis of the congenital hand malformations is usually difficult and delayed. Thus, more diagnosis methods are required.

In addition to the variable phenotypes of congenital hand malformations, its genetic heterogeneity creates significant challenges for diagnosis. Syndactyly, a common disorder of congenital hand malformations, can be inherited as an autosomal dominant (AD), autosomal recessive (AR), and X-linked recessive (XR) model^6,7^. In addition, the number of identified genes associated with congenital hand malformations has been growing, and research involving the etiology of congenital hand malformations has been flourishing in the past decade. Although the functions of some of these genes have been extensively studied, it is still difficult to establish a precise genotype-phenotype correlation.

Genetic variations play an important role in congenital hand malformations. Determining the causative variants in patients affected by congenital hand malformations provides them with a number of benefits; for example, it can supply appropriate genetic counseling about recurrent risk and an accurate prognosis of the clinical course of the disease^8^. It is also critical for prenatal diagnosis because serious fetal hand deformities can have a strong impact on family and social life, and a necessary treatment plan should be undertaken before birth^2,9^. Moreover, the molecular diagnosis of patients evaluates the etiology of congenital hand malformations in clinics and lays the foundation for future inclusion in clinical trials based on gene therapy.

In recent years, the development of next-generation sequencing (NGS) has allowed to screen a large number of genes with a high sensitivity. Compared with the time-consuming, expensive, and gene-by-gene traditional analysis method, NGS is a faster, cheaper, and more efficient method for mutation screening. Recent studies have demonstrated that NGS has been successfully used to diagnose heterogeneous diseases^8,10–13^.

In this study, we applied a targeted NGS to screen 248 genes known to be associated with genetic skeletal disorders in three Chinese families with congenital hand malformations. We identified three pathogenic mutations in the *GJA1*, *ROR2* and *TBX5* genes. Our results support the application of targeted NGS in clinical diagnosis.

## Results

### Clinical description

**Figure 1:**
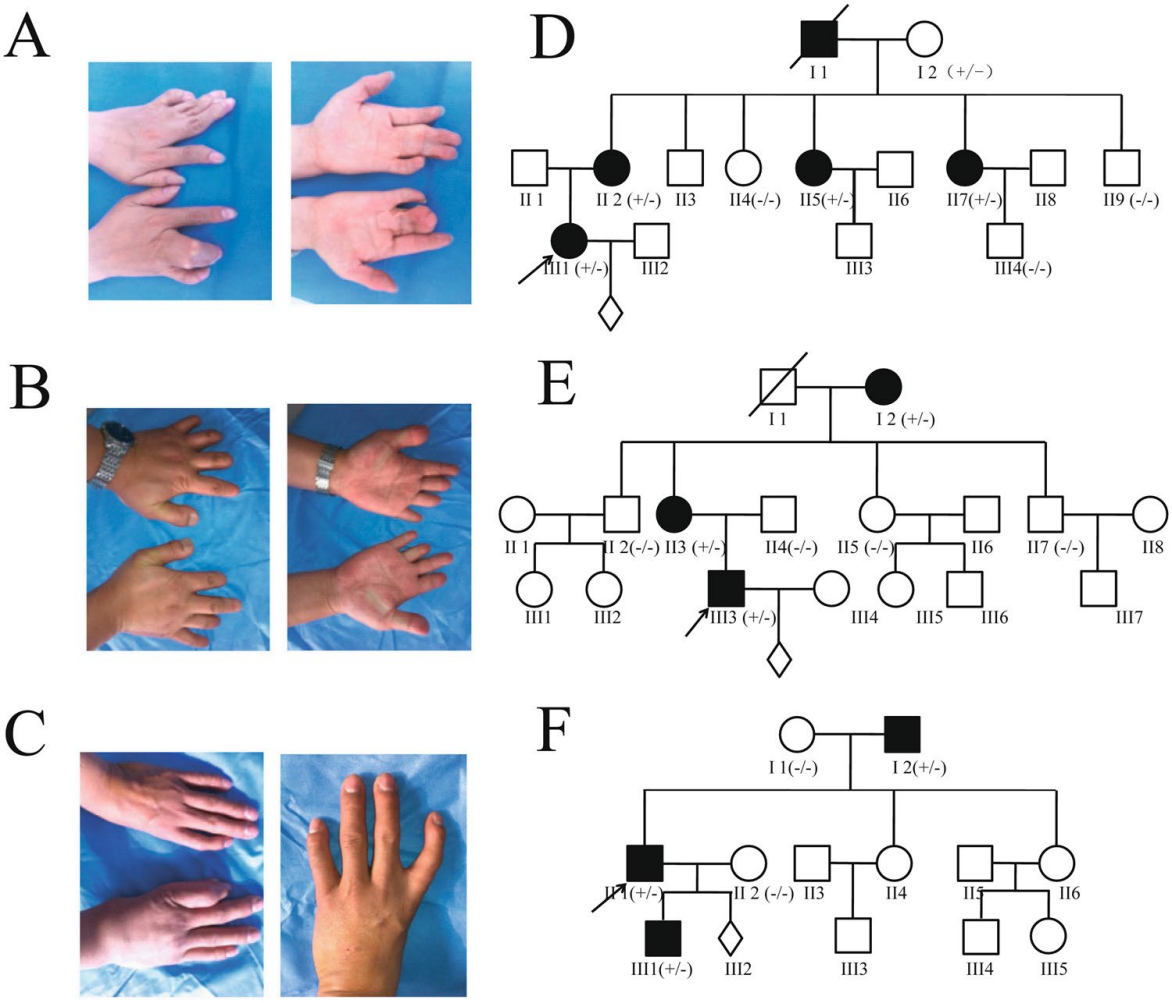
Clinical phenotypes and pedigrees of the cases. (**A**) The proband showed bilateral syndactyly between third and fourth fingers in Case 1. (**B**) The proband of Case 2 showed bilateral hypoplasia of the distal and middle phalanges of fingers 2–5, nail hypoplasia and the absence of nails on some fingers. (**C**) Proband of Case 3 was afflicted with an absent thumb. (**D**–**F**) Pedigrees of Case1, Case2 and Case3. Solid boxes and circles indicate affected individuals. The proband is marked with a black arrow. Symbols +/− and −/− represent heterozygous mutation and wild-type genotype respectively.

Three patients with congenital hand malformations and their families were recruited from Henan Provincial People’s Hospital, Henan, China. All of the patients were characterized by visible hand deformities such as SD, BD, and ektrodactylia (Fig. 1A–C, and Supplementary Figs S1 and S2). Pedigrees of the cases recruited in this study are shown in Fig. 1D–F. Available family members were recruited to assist in the analysis of pathogenic variations.

### Identification of candidate mutations by targeted NGS

A total of 248 genes known to be associated with genetic skeletal disorders were the target genes in this study, including genes involved in hand malformations (Supplementary Table S1). All exons, splicing sites, and the immediately adjacent introns of these genes were included in the NGS panel.

In general, at least 500 Mb of clean data was obtained from the raw data for each patient. The average sequencing depth was more than 246X, and the mean coverage of target regions was greater than 99.6%. Coverage of the target base for the N10 and N20 was more than 96.2% and 93.0%, respectively. An overview of the NGS data is listed in Supplementary Table S2.

In order to avoid false negatives in clinical diagnosis, we filter and retain the variants with allele frequency less than 5%. After annotation and filtration, there were three candidates of pathogenic mutations, including a missense mutation, a nonsense mutation and a splice-site mutation. The heterozygous mutation-c.388 A > T (p.I130F)-of the *GJA1* gene, the heterozygous mutation-c.2247 G > A (p.W749X)-of the *ROR2* gene, and the heterozygous splice-site mutation-c.663 + 1 G > C- of the *TBX5* gene were found in Case 1, Case 2, and Case 3, respectively (Supplementary Fig. S3).

To validate the positive correlation of these mutations with diseases, Sanger sequencing was employed to detect mutations in patients and their available family members. The results showed three mutations in different genes with the mode of autosomal dominant inheritance (Fig. 1).

### Characterization of candidate mutations

**Figure 2:**
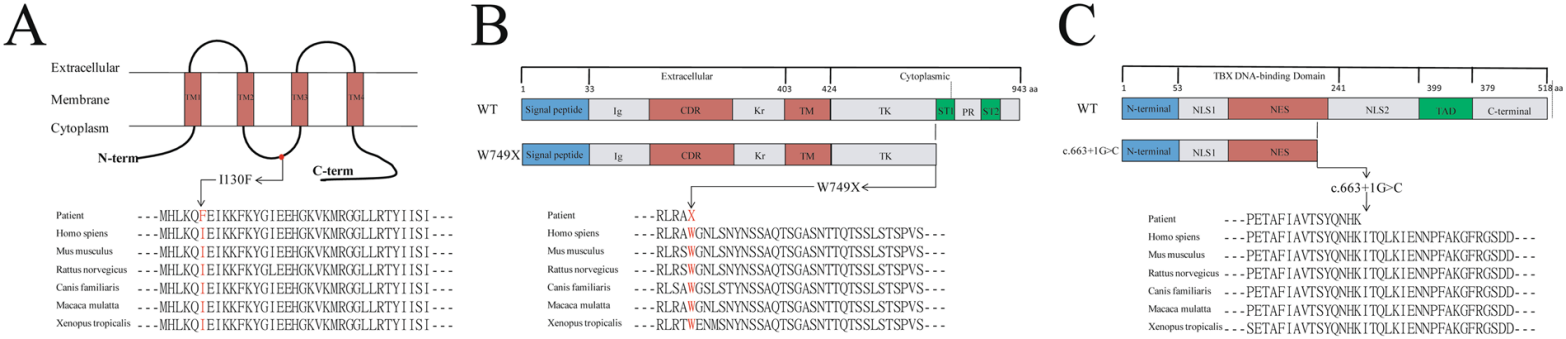
Mutation analysis of c.388 A > T (p.I130F) in the *GJA1* gene, c.2247 G > A (p.W749X) in the *ROR2* gene and c.663 + 1 G > C in the *TBX5* gene. (**A**) Domain structure of Connexin 43 (Cx43) and protein conservation analysis of the mutations across multiple species. The Cx43 mutations indicated as a red dot in the intracellular loop domain. (**B**) Diagram of the ROR2 molecule and protein conservation analysis of the mutations across multiple species. (**C**) Schematic representation of the TBX5 protein and protein conservation analysis. NLS1 = nuclear localization segment 1, NLS2 = nuclear localization segment 2, NES = nuclear export segment.

The only one missense mutation of c.388 A > T (p.I130F) in GJA1 gene was predicted pathogenic mutation by some of the software tools (Supplementary Table S4). The missense mutation was located in exon 2 and resulted in the replacement of isoleucine with phenylalanine. Conservative analysis of the protein sequence among various species showed the region, including the mutation p.I130F, was highly conserved (Fig. 2A). The SIFT, PoluPhen, and MutationTaster predicted that the c.388 A > T (p.I130F) mutation was a pathogenic mutation, with values of 0.08, 0.824, and 1, respectively. Although the c.388 A > T (p.I130F) mutation was not reported in several databases (Human Gene Mutation Database (HGMD), the Exome Aggregation Consortium (ExAC) database, and the 1000 Genomes database), the c.389 T > C (p.I130T) mutation was reported to be a pathogenic mutation in the HGMD database^14^. Taken together, the c.388 A > T (p.I130F) mutation was considered pathogenic in Case 1.

In Case 2, a heterozygous nonsense mutation, c.2247 G > A (p.W749X), was detected in *ROR2* gene. The mutation caused a premature stop codon and resulted in the loss of 195 amino acids. Conservative analysis of the protein sequence showed the amino acid (Tryptophan) was highly conserved among various species (Fig. 2B). Moreover, both the c.2247 G > A (p.W749X) and c.2246 G > A (p.W749X) mutations had been reported as pathogenic mutations in the HGMD database^15,16^.

A splice-site mutation of the *TBX5* gene was detected in Case 3. The c.663 + 1 G > C mutation was a canonical splice site mutation, located in sixth intron. Conservative analysis of the genomic DNA sequencing showed that the base, located in a splice-site, was highly conserved. To predict whether the mutation changed the transcriptional profile, NetGene 2 was employed to evaluate the effect of the mutation. The result showed that the mutation was caused a loss of splice-site. MutationTaster predicted that the mutation was pathogenic, with a value of 1. Moreover, Sanger sequencing results also showed that the mutation had a mode of autosomal dominant inheritance. In summary, these experiments strongly suggest the splice-site of c.663 + 1 G > C in the *TBX5* gene was the causative mutation in Case 3.

## Discussion

There are many syndromes that are associated with congenital hand malformations^17^. The significant phenotype and genetic heterogeneity of congenital hand malformations is a great challenge for clinical diagnosis and genetic counseling. The traditional detection methods for individual genes are time-consuming and expensive. In this study, targeted NGS was performed to screen 248 genes involved in genetic skeletal disorders, including genes associated with congenital hand malformations. We identified pathogenic mutations in three large Chinese families with congenital hand malformations, and these pathogenic mutations belonged to known hand deformity genes, including *GJA1*, *ROR2* and *TBX5*.

The missense mutation of c.388 A > T (p.I130F) found in Case 1 is located in the *GJA1* gene, which is a member of the connexin gene family. *GJA1* encodes the gap junction protein alpha-1 (Connexin 43, Cx43), which is a major component of gap junctions in osteoblasts, osteocytes, osteoclasts and chondrocytes^18^. Syndactyly type III (SD3) is characterized by bilateral complete syndactyly between the fourth and fifth fingers, with occasional involvement of the third fingers^19^. In Case 1, the proband (III 1) showed bilateral syndactyly between the third and fourth fingers; however, the proband’s mother (II 2) showed bilateral syndactyly between the fourth and fifth fingers (Fig. 1A and Supplementary Fig. S1). SD3 is inherited in an autosomal dominant pattern with incomplete penetrance^7^. The Sanger sequencing results showed that the c.388 A > T(p.I130F) mutation was inherited with an autosomal dominant model in Case 1. Although the phenotype and genetic results suggested the disease was SD3, another syndrome, oculodentodigital dysplasia (ODDD), was considered the clinical diagnosis of this case. ODDD is a rare genetic disorder usually inherited in an autosomal dominant manner, and SD3 has been reported to occur as a part of ODDD^20,21^. In addition to the phenotypes of SD3, the affected members showed camptodactyly, short and narrow palpebral fissure, a narrow nose, and hypoplastic enamel (Supplementary Fig. S1), which are characteristics of ODDD^22^. According to the molecular results and phenotype, we reclassified the syndrome of Case1 as ODDD. The reclassification according to molecular diagnosis is crucial for accurate genetic counseling and evaluates the knowledge in the clinic.

Brachydactyly type B (BDB), one type of BD, is the most severe of the brachydactylies and is characterized by hypoplasi of distal phalanges and nails^23,24^ (Fig. 1B and Supplementary Fig. S2). The c.2247 G > A (p.W749X) mutation of the *ROR2* gene in Case 2 was the pathogenic mutation of brachydactyly tape B1 (BDB1), which is a subtype of BDB. *ROR2*, located on chromosome 9q22, encodes the orphan receptor tyrosine kinase (RTK)-like orphan receptor 2 (ROR2). The structure of ROR2 is composed of an extracellular region, a transmembrane domain, and an intracellular domain. The extracellular region associated with protein-protein interaction, which contains an immunoglobulin-like domain (Ig), a cysteine-rich domain (CRD), and a kringle domain (KD). The intracellular region includes a trysine kinase domain (TK), two serine/threonine-rich domains (ST), and a proline-rich domain (PR)^16,25,26^. The nonsense mutation found in Case 2 resulted in a premature stop codon and caused the defects of the PR domain and two ST domains (Fig. 2B). Oldridge M *et al*. reported that the deletion of ROR gene on an allele could not cause BDB1; however, the intragenic mutations resulted in BDB1^16^. In this case, the truncated protein was the causative etiology.

Mutations of the *TBX5* gene caused Holt-Oram syndrome (HOS), which is a rare syndrome and is characterized by a malformation of the upper limbs and variable cardiac defects^27,28^. The mutation of c.663 + 1 G > C found in Case 3 was a splice-site mutation, which resulted in the incorrect transcription of the TBX5 protein and caused the changes of the second nuclear localization segment (NLS), a transactivation domain, and the C-terminal region^29^ (Fig. 2C). The truncated protein is unable to perform accurate nuclear localization and is unable to bind with other proteins to active downstream signals. According to the molecular diagnosis results, we also supplied a prognosis for this family. The proband (II 1) of Case 3 is not only affected with an absent thumb but also has an atrial septal defect, while his 3-year-old son (III 1) presented with a triphalangeal thumb. During genetic counseling, we recommended a heart test to be performed on the proband’s son. The results revealed that the proband’s son is also affected with a mild atrial septal defect, and pediatric cardiothoracic surgeons guided further treatments. In this case, we were not only able to detect the pathogenic mutation but also predicted the unknown phenotype due to accurate molecular diagnosis.

Accurate molecular diagnosis is crucial for classification in clinical diagnosis and is also important for individual treatment. Recently, NGS was widely used in diagnosis of single gene inheritance diseases, heterogeneous diseases and cancers^30–32^. In this study, targeted NGS was successfully used to detect the causative variations in three large Chinese families with congenital hand malformations, which are highly phenotypic and genetically heterogeneous. However, it is important to be aware of NGS limitations, such as miss detections of small insertions/deletions, trinucleotide repeats and CNV variations. Targeted gene panels are typically less expensive than whole exome sequencing, but go out of date as new causal genes are discovered for relevant phenotypes. Whole exome sequencing covers all annotated coding exons and will be widely used in future clinical diagnostics.

In conclusion, targeted NGS was employed to screen 248 genes involved in genetic skeletal disorders, and three pathogenic mutations located in *GJA1*, *ROR2* and *TBX5* were detected in three large Chinese families with congenital hand malformations. To our knowledge, this is the first report of the c.388 A > T (p.I130F) mutation in the *GJA1* gene and the c.663 + 1 G > C mutation in the *TBX5* gene, while the c.2247 G > A (p.W749X) mutation in the *ROR2* gene was verified as a pathogenic mutation in Chinese population. These findings expand the mutation spectrum and the population specific variants frequency. This is also the first report that the same panel of targeted NGS was employed to perform molecular diagnosis of different subtypes of congenital hand malformations. Our study supported the application of a targeted NGS panel as an effective tool to detect the genetic cause for heterogeneous diseases in clinical diagnosis.

## Methods

### Targeted NGS approach

The probes used in this study were RNA and is about 120 bp. Targeted genes were chosen according to OMIM database and were designed by the MyGenostics company (Beijing, China).Genomic DNA was extracted from the peripheral blood lymphocytes of probands and their family members using the Qiagen genomic DNA isolation kit (Qiagen, Hilden, Germany) according to the manufacturer’s instructions. The genomic DNA of the probands was quantified by NanoDrop (Thermo, Waltham, USA). Informed consent was obtained from all subjects. This study was approved by the Ethics Committee of Henan Provincial People’s Hospital and conducted in accordance with relevant guidelines and regulations.

Libraries of probands used in targeted NGS were prepared as described previously^33^. 3 µg of genomic DNA of each proband was fragmented by nebulization, end-repaired, an ‘A’ was ligated to the 3′-end, the fragments were marked by Illumina adapters, and the size selected was performed aiming for a 350–400 base pair product. Then, the final products were amplified by PCR and validated using the Agilent Bioanalyzer.

The amplified DNA was captured with Target Enrichment System (MyGenostics, Beijing, China) according to the manufacturer’s protocol. To perform the hybridization, 1 µg of DNA library was mixed with Buffer BL and GenCap probe (MyGenotics, Beijing, China) to perform pre-hybridization, and Buffer HY (MyGenotics, Beijing, China) was added to the mixture for further hybridization. After hybridization, the target DNA was bound to MyOne beads (Thermo, Waltham, USA) and eluted with Buffer Elute. Then, the eluted DNA received final PCR and purification. The enrichment libraries were sequenced on Illumina HiSeq. 2000 sequencer (Illumina, San Diego, USA) for paired read 100 bp according to the manufacturer’s protocol.

### Bioinformatics analysis

Clean reads were generated from raw data by removing the adapters and the low quality reads. The BWA program package was used to align the filtered reads to the human reference genome, and the GATK software package was employed to recalibrate quality scores and realign to reference. Sequence Alignment/Map tools 3 (SAMtools 3) was used to remove the duplicated reads. The uniquely mapped reads were used for variation detection.

Single nucleotide variants (SNVs) were detected with the GATK UnifiedGenotyper, and the variants were filtered by GATK VariantFiltration. Indels (insertions and deletions) were called by GATKIndelGenotyper V2 Annotation. Variants were annotated by an in-house developed bioinformatics tool with RefSeq (hg19, from UCSC) and UCSC annotation.

Several databases (dbSNP138, 1000 Genomes, and an in-house Asia database) were used to filter the SNPs/indels, which showed >5% frequency in these databases. Several bioinformatic software tools (SIFT, PoluPhen and MutationTaster) and several online websites (InterVar and Clinvar) were employed to predict the probable pathogenic mutation.

### Sanger sequencing

Sanger sequencing was used to confirm these candidate pathogenic mutations. Primers were designed to amplify the mutations and flanking sequences (Supplementary Table S3). PCR products were purified with a gel extraction kit (Promega, Madison, USA) according to the manufacturer’s protocol, and sequenced on an ABI 3130 sequencer (Thermo, Waltham, USA). The sequencing results were analyzed by sequence analysis software.

## Electronic supplementary material

Supplementary materials
