# Supplementary material for: Targeted next-generation sequencing-based molecular diagnosis of congenital hand malformations in Chinese population
Source: Sci Rep. 2018 Aug 24;8:12721. doi: 10.1038/s41598-018-30940-6 (PMC6109141; doi:10.1038/s41598-018-30940-6)
Supplement: Supplementary file 1 — Supplementary materials [file 41598_2018_30940_MOESM1_ESM.pdf]

Targeted next-generation sequencing-based molecular diagnosis of congenital hand malformations in Chinese population

Litao Qin[1], Guiyu Lou[1], Liangjie Guo[1], Yuwei Zhang[1], Hongdan Wang[1], Li Wang[1], Qiaofang Hou[1], Hongyan Liu[1], Xichuan Li[2], Shixiu Liao[1]

[1]Medical Genetic Institute of Henan Province, Henan Provincial Key Laboratory of Genetic Diseases and Functional Genomics, Henan Provincial People's Hospital, People's Hospital of Zhengzhou University, Zhengzhou, Henan, China.

[2]Department of Immunology, Tianjin Medical University, Tianjin, China.

Correspondence to: Shixiu Liao. **email:** litao_qin@163.com, Xichuan Li. **email:** lixichuan@tmu.edu.cn

Supplemental Data

Supplementary Table S1 Gene list of NGS panel

ACAN	ACP5	ACVR1	ADAMTSL2	AGA	AGPS	ALPL
ALX1	ALX3	ALX4	AMPD3	ANKH	ANO5	ANTXR2
ARSB	ARSE	ASPN	ATP6V0A2	B4GALT7	BMP1	BMP2
BMPER	BMPR1B	CA2	CANT1	CASR	CC2D2A	CDH3
CEP290	CHST14	CHST3	CIAS1	ClCN5	CLCN7	COG1
COL10A1	COL11A1	COL11A2	COL1A1	COL1A2	COL2A1	COL9A1
COL9A2	COL9A3	COMP	CREBBP	CRTAP	CTSK	CUL7
CXORF5	DDR2	DHCR24	DHODH	DLL3	DLX3	DMP1
DTDST	DYM	DYNC2H1	EBP	EFNB1	EIF2AK3	ENPP1
EP300	ESCO2	EVC	EVC2	EXT1	EXT2	FAH
FAM20C	FAM58A	FBLN1	FBN1	FBN2	FBXW4	FERMT3
FGF10	FGF23	FGF9	FGFR1	FGFR2	FGFR3	FKBP10
FLNA	FLNB	FMN1	FOXE1	FRZB	FUCA1	FUCA2
GALNS	GALNT3	GDF5	GDF6	GJA1	GLB1	GLI3
GNAS1	GNPAT	GNPTAB	GNPTG	GNS	GORAB	GPC6
GREM1	GUSB	HDAC4	HES7	HGSNAT	HLXB9	HOXA11
HOXA13	HOXD13	HPD	HPGD	HSPG2	HSS	ICK
IDS	IDUA	IFITM5	IFT122	IFT80	IHH	IKBKG
IL1RN	LBR	LEMD3	LEPRE1	LFNG	LIFR	LMBR1
LMNA	LMX1B	LPIN2	LRP4	LRP5	MANA	MANB
MATN3	MESP2	MGP	MKS1	MMP13	MMP2	MMP9
MSX2	MYCN	MYH8	NAGLU	NEK1	NEU1	NF1
NFIX	NIPBL	NKX3-2	NOG	NPPC	NPR2	NSD1
NSDHL	OBSL1	OPG	OSTM1	PAPSS2	PCNT2	PEX7
PHEX	PHF6	PIGV	PITX1	PLEKHM1	PLOD2	PLOD3
POLR1C	POLR1D	POR	PPGB	PPIB	PTH1R	PTHLH
PTHR1	PTPN11	PYCR1	RAB23	RASGRP2	RECQL4	RMRP
ROR2	RPGRIP1L	RUNX2	SALL1	SALL4	SBDS	SEDL
SERPINF1	SERPINH1	SH3BP2	SH3PXD2B	SHH	SHOX	SLC17A5
SLC26A2	SLC34A3	SLC35D1	SLC39A13	SLCO5A1	SMARCAL1	SOST
SOX9	SP7	SULF1	SUMF1	TAT	TBCE	TBX15
TBX3	TBX4	TBX5	TBXAS1	TCIRG1	TCOF1	TGFB1
TGFBR1	TGFBR2	THPO	TIMM8A	TMEM16E	TMEM38B	TMEM67
TNFRSF11A	TNFRSF11B	TNFSF11	TP63	TREM2	TRIP11	TRPS1
TRPV4	TWIST1	TYROBP	WDR35	WISP3	WNT1	WNT3
WNT7A	WTX	ZMPSTE24				

Supplementary Table S2 Overview of probands' NGS data in three cases

	Raw data (Mb)	Clean data (Mb)	Coverage of target region	Average sequencing depth on target	N10	N20
**Case 1**	840.69	837.49	99.70%	570.95	97.00%	93.30%
**Case 2**	1043.25	1032.37	99.60%	683.67	97.10%	93.70%
**Case 3**	545.36	538.28	93.00%	246.62	87.8%	80.9%

Supplementary Table S3 Primers used in Sanger sequencing

Case 1	*GJA1*-388 F	5' GTTCTATGTGATGCGAAAGGA 3'
	GJA1-388 R	5' AAGGCCACCTCAAAGATAGAC 3'
Case 2	*ROR2*-2247 F	5' GTCCCGCCTGGGTGTATG 3'
	ROR2-2247 R	5' GGCGTTGCTCACATTGCT 3'
Case 3	*TBX5*-663 F	5' AAAGCGGATGAAAATAATGG 3'
	TBX5-663 R	5' GCCATTCAGAGGAGCAAAG 3'

Supplementary Table S4 Overview of filtered and remained mutations in case 1

Gene_symbol	Transcript	Exon	Coding	Protein	1000	Inhouse	snp138	SIFT	PolyPhen_2	MutationTaster	InterVar	Clinvar
AMPD3	NM_001172431	exon5	c.454G>T	p.V152L	0.0032	-	rs117706710	0.04	0.986	1	Likely benign	Likely benign
ESCO2	NM_001017420	exon3	c.335C>G	p.T112S	-	-	-	0.75	0.005	1	Likely benign	Likely benign
FAM20C	NM_020223	exon10	c.1681G>A	p.V561M	0.02	0.0093209	rs145750007	0.13	0.607	0.61	Benign	Benign
GJA1	NM_000165	exon2	c.388A>T	p.I130F	-	-	-	0.08	0.824	1	Likely pathogenic	UNK
MGP	NM_000900	exon1	c.23C>T	p.A8V	0.01	0.0079893	rs142330429	0.02	0.018	1	Benign	Benign|Likely benign
PCNT	NM_006031	exon39	c.8884G>A	p.G2962S	-	-	-	0.76	0.003	1	Likely benign	UNK
PEX7	NM_000288	exon7	c.695G>A	p.R232Q	0.0018	0.0013316	rs191969418	0.83	0.004	1	Uncertain significance	Benign|Likely benign
ROR2	NM_004560	exon9	c.2212C>T	p.R738C	0.0014	0.0019973	rs56231927	0.02	1	1	Likely benign	UNK
TAT	NM_000353	exon12	c.1270G>A	p.E424K	-	-	-	0.83	0.003	0.997	Uncertain significance	UNK
TRIP11	NM_004239	exon15	c.5086G>A	p.E1696K	0.02	0.0156458	rs80200454	0.16	0.043	0.998	Benign	Likely benign|Likely benign
TRIP11	NM_004239	exon11	c.1904C>G	p.S635C	0.02	0.0226365	rs59635749	0.05	0.995	1	Benign	UNK

To provide better sensitivity for detecting deleterious SNPs， the SIFT、PolyPhen-2 and Mutation Taster scores are ＜ 0.1, > 0.85 and > 0.5 respectively.

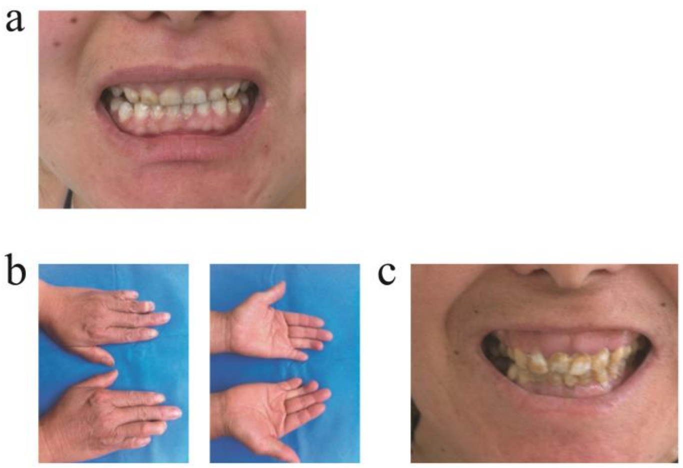

Supplementary Figure S1 Clinical phenotypes of proband and her mother in Case 1. (a) Proband shows narrow nose and hypoolastic enamel. (b, c) Proband's mother shows the similar pthenotypes with proband, while she showed bilateral syndactyly between fourth and fifth fingers.

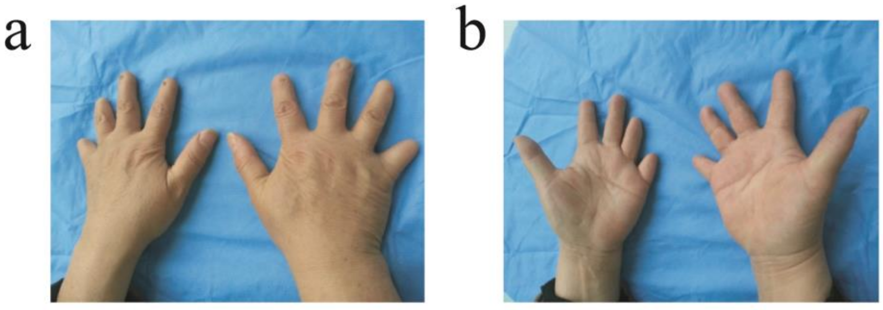

Supplementary Figure S2 Photographs of the proband's mother's hands in Case 2. She showed shortened or absent distal phalanges on fingers 2–5.

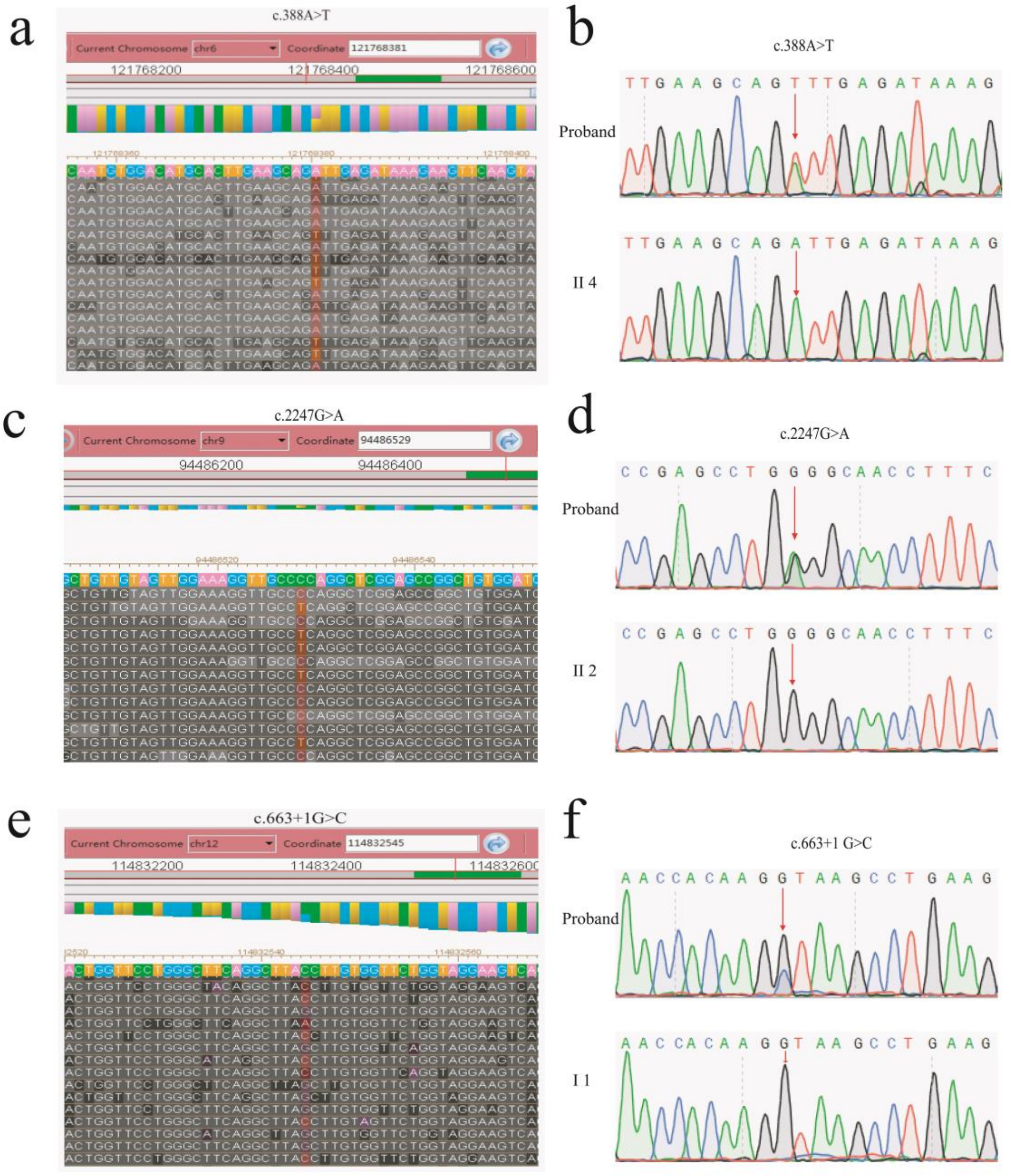

Supplementary Figure S3 Identification of mutations in the cases. a, c, e. Three mutations: c.388A>T (p.I130F) in the *GJA1* gene, c.663+1G>C in the *TBX5* gene and c.2247G>A (p.W749X) in the *ROR2* gene were detected by targeted NGS in the three cases. **b, d, f.** Sanger sequencing verified the mutation in family members of different cases.
